# Transition readiness among finnish adolescents with juvenile idiopathic arthritis

**DOI:** 10.1186/s12969-023-00938-0

**Published:** 2023-12-21

**Authors:** Katriina Mikola, Katariina Rebane, Hannu Kautiainen, Kristiina Aalto

**Affiliations:** 1 New Children’s Hospital, Pediatric Research Center, University of Helsinki and Helsinki University Hospital, Stenbackinkatu 9, 00290 Helsinki, Finland; 2 Kuopio University Hospital, Primary Health Care Unit Kuopio, Pohjois-Savo, Finland; 3 Folkhälsan Research Center, Helsinki, Finland

**Keywords:** Juvenile idiopathic arthritis, Transition, Outcome, Self-management

## Abstract

**Background:**

With chronic diseases, the responsibility for care transfers to adult clinics at some point. Juvenile idiopathic arthritis (JIA) is the most common persistent rheumatic condition in children. A successful transition requires sufficient self-management skills to manage one´s chronic condition and all the tasks involved. In this study, we evaluated transition readiness in Finnish patients with JIA. We aimed to find practical tools to support a successful transition and to study the possible consequences of an unsuccessful transition.

**Methods:**

The usefulness of a specific questionnaire, which was administered to 83 JIA patients, was evaluated in this study. We also gathered information from their first adult clinic visit to assess the success of their transition and its relation to disease activity.

**Results:**

In 55 (71%) patients, the transition was estimated to be successful. We were able to determine a cut-off score in the questionnaire for a successful transition: the best estimate for a successful transition is when the score is 24 or more. At the first adult clinic visit, an unsuccessful transition was evident in its effect on disease outcome. If the transition was defined as successful, the DAS28 was better.

**Conclusion:**

We found the questionnaire to be a useful tool for evaluating transition readiness. Determination of a successful transition helped us identify those adolescents who needed more profound support to improve their self-management skills and thus enhance their transition process. An unsuccessful transition was shown to negatively impact on disease outcomes.

## Background

Juvenile idiopathic arthritis (JIA) is the most common persistent rheumatic condition in children [1]. By nature, it is chronic; in a Nordic cohort, at a time point 18 years after diagnosis, the disease was still active in 46% of patients, with 15% being treated with synthetic disease-modifying antirheumatic drugs (sDMARDs) and 19% with biologics (bDMARDs) [2].

As adolescents with JIA grow up, their disease is no longer monitored in a paediatric clinic, and the responsibility for their care is moved to an adult clinic. However, this transition involves more than just the actual point of transfer, it begins in early adolescence and will later involve the adult clinic team as well [3]. In a systemic review involving a number of chronic diseases, a structured transition was generally found to promote patients’ overall outcomes in many aspects of their transitions [4]. It has been shown that patients with JIA benefit from a planned transition; for example, the drop-out rates from care diminish [5].

JIA also involves many comorbidities [6], which increase the burden of this chronic disease [7]. One of the most common comorbidities is JIA-related uveitis [8, 9]. Having a chronic physical condition also increases the risk of mental disorders in youth [10–12]. These issues place additional demands on the transition. Sufficient self-management skills form the basis of a successful transition [13]. There are many types of practice that can enhance transition readiness and improve the self-management skills of these adolescents [14].

So far, there has not been an appropriate questionnaire to evaluate the transition readiness in Finnish patients with JIA. The purpose of our study was to evaluate the self-management skills and transition readiness in Finnish patients with JIA and to estimate the usefulness and applicability of the specially designed PETRA questionnaire (**Pe**diatric **t**ransition **r**eadiness to **a**dult care) in the Finnish health care system.

Our aim was to find practical tools to support a successful transition and to study the possible consequences of an unsuccessful transition on disease outcomes. Our aim was to improve the transition process with this pilot PETRA questionnaire and thus be able to support adolescents and their families more effectively.

## Methods

This was a retrospective, real-life study based on our clinical practices in the transition of patients with JIA. PETRA questionnaire was developed and inspired by a Canadian Good 2 Go questionnaire (www.sickkids.ca/en/patients-visitors/transition-adult-care). This pilot PETRA questionnaire evaluates several aspects of self-management, such as independence in disease management (medication, appointments, pain control), everyday life (school, future educational plans, mental support, exercise, sexual health), and substance abuse. The paper version of the questionnaire was in routine use in our paediatric rheumatology clinic in the Hospital District of Helsinki and Uusimaa (HUS) between June 2011 and December 2013. Due to changes in the electronic patient record system, the use of this paper version remained temporary, while the transition procedures have remained essentially unchanged in our clinic. Based on the clinician’s evaluation, the questionnaire was given to adolescents who were planned to be transferred to an adult clinic, comprising patients with an ongoing disease activity and who were on systemic antirheumatic medication. Patient with disease on remission without medication [15] was not included. Altogether, 83 patients received and filled in the questionnaire as part of a routine rheumatological visit at the paediatric site.

In our final analysis, we used 13 questions, selected by consensus by an expert research team, based on opinions and psychometric evaluation, with three answer options (yes = 2, partly = 1, or no = 0). Higher scores indicated better readiness.

We also gathered information about the patients from the medical records of their first adult visit. The patients’ reported outcomes were measured using the Health Assessment Questionnaire (HAQ), the visual analogue scales (VASs) for pain [16], the global assessment of well-being, and their disease activity scores (DAS28). The physician-reported global assessment of disease activity was measured on a 21-numbered VAS scale [17]. Information about the social participation (including education, employment status and leisure-time activity) and the health behaviour (including smoking and physical activity) of the patients was also gathered. Non-restricted social participation involved engagement in studying, working, maternity leave, or military service [18].

To define the success of the transition, information was collected from both the paediatric and adult patient records. Based on a consensus of the research team’s expert opinions, with adjustments for the practices of the Finnish healthcare system, the key elements for a successful transition were defined as: [1] the patient was able to attend the first visit at the adult care centre independently, [2] the first visit took place as scheduled without extra communication, and [3] the medication was carried out as agreed at the last paediatric visit.

According to the transition practices of our paediatric rheumatology clinic in HUS, we do not transfer all patients to the adult site [19, 20], but only those with active disease or on ongoing medication. If the disease activates later, these patients will be referred to the adult rheumatology clinic, for example, from primary health care, student, or occupational health care. A special rheumatological transition clinic is provided at the adult site [20], and special attention is paid, for example, to avoid dropping out of follow-up. If a patient does not appear for a visit as planned, the designated nurse will contact him or her.

## Analyses

Data were presented as means with standard deviation (SD) and as counts with percentages. The Kaplan–Meier method was used to estimate the crude cumulative transition rate. Receiver operating characteristic (ROC) curves were used to determine an optimal cut-off value of PETRA questionnaire for discerning successful transition. We defined the best cut-off value as the value with the highest accuracy that maximized Youden’s index (sensitivity + specificity − 1). In general, an AUC of 0.5 suggests no discrimination (that is, the ability to distinguish those patients who had successful of transition or failed to transition based on the test), 0.7 to 0.8 is considered acceptable, 0.8 to 0.9 is considered excellent, and more than 0.9 is considered outstanding [21]. The area under the curve (AUC), sensitivity, specificity, and odds ratio (OR) were calculated; 95% confidence intervals were obtained by bias corrected bootstrapping (5000 replications). We also assessed floor and ceiling effects for items and total score by calculating the proportion of patients who obtained the lowest or highest scores. The difference between the transfer groups for DAS28 values was evaluated using a t-test. The Stata 17.0 (StataCorp LP; College Station, Texas, TX, USA) statistical package was used for the analysis.

## Results

The clinical characteristics of the patients are shown in Table 1.

**Table 1:** The clinical characteristics of the patients at the time when they filled in the questionnaire at the paediatric site and, later, at their first adult clinic visit

	Questionnaire filled in at paediatric site N = 83	First adult clinic visit N = 77
Female, n (%)	59 (71)	53 (69)
Age at onset, years, mean (SD)	8.9 (5.2)	
Age at visit, years, mean (SD)	14.5 (0.9)	17.1 (1.5)
JIA category* n (%)		
Polyarthritis, RF+	6 (7)	6 (8)
Polyarthritis, RF-	26 (31)	26 (34)
Juvenile psoriatic	5 (6)	3 (4)
Enthesitis-related	5 (6)	5 (6)
Undifferentiated	2 (2)	2 (3)
Persistent oligoarthritis	23 (28)	22 (29)
Extended oligoarthritis	16 (19)	13 (29)
Remission**, n (%)	61 (73)	54 (71)
Uveitis, n (%)	23 (28)	20 (26)
Physician’s global VAS 0-100, mean (SD)	4.3 (8.8)	4.8 (12.0)
ESR, mean (SD)	7.3 (7.6)	7.6 (6.9)
Active joints, mean (SD)	0.5 (1.1)	0.4 (1.0)
Patient’s pain VAS 0-100, mean (SD)		17.3 (22.2)
Patient’s global VAS 0-100, mean (SD)		12.6 (19.6)
DAS28, mean (SD)		1.57 (0.79)
Systemic medication, n (%)	72 (87)	63 (82)
Synthetic DMARDs	68 (82)	54 (70)
Biologic DMARDs	31 (37)	33 (43)
Systemic steroids	19 (23)	11 (14)
Smoking, n (%)		3 (12)
Non-restricted social participation, n (%)		75 (97)

*According to the ILAR classification criteria [31]. **According to Wallace’s preliminary criteria [15]

RF = rheumatoid factor, VAS = visual analogy scale, ESR = erythrocyte sedimentation rate, DMARD = disease-modifying antirheumatic drug

**Fig. 1:**
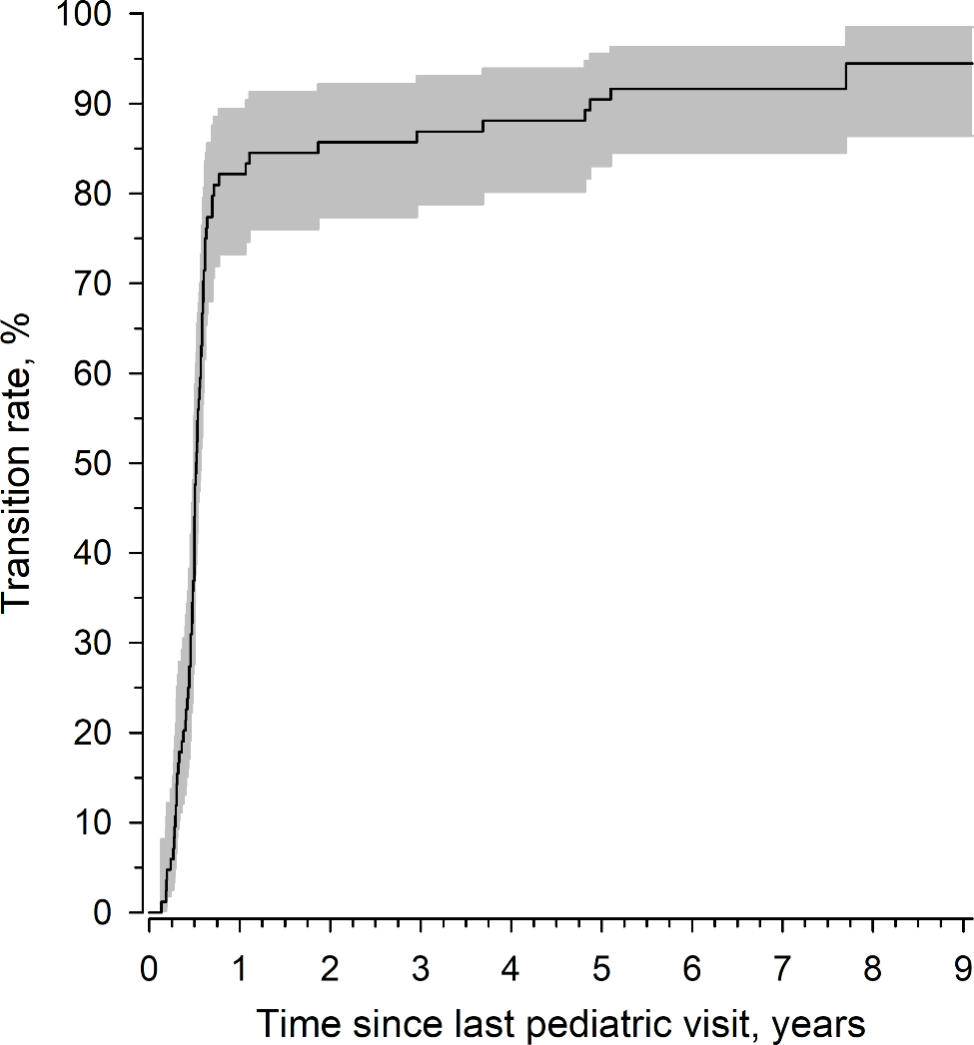
Cumulative transition rate from paediatric to adult care in patients with JIA. Kaplan–Meier estimates of the cumulative transition. The grey area represents a 95% confidence interval

Sixteen of the 83 patients who filled out the questionnaire did not need the transition to the adult clinic when they reached the transition age of 16 years. Nevertheless, 11 of these 16 arrived at the adult site during the follow-up period (Fig. 1). Therefore, altogether, only five patients were not transferred during this observation period.

The mean score from the transition readiness questionnaire was 22.5 (SD 2.2) and the median (IQR) was 23 (21.25). Ten patients (10%) received the maximum score 26.

Table 2 shows the individual questions contained in the PETRA questionnaire. Overall, the readiness score was satisfactory, but the questions regarding independence revealed the lowest level of skills.

**Table 2:** The PETRA questionnaire and its descriptive values

Questions	Range	Mean (SD)	Floor effect N (%)	Ceiling effect N (%)
I can describe my disease and my symptoms.	0–2	1.73 (0.47)	1 (1)	62 (75)
I can spend time alone during the control visits and take part in discussion.	0–2	1.41 (0.61)	5 (6)	39 (47)
I know the names of my medicines and their doses.	0–2	1.72 (0.50)	2 (2)	62 (75)
I know how to relieve pain.	0–2	1.70 (0.49)	1 (1)	59 (71)
I am in charge of taking my medications on my own.	0–2	1.39 (0.68)	9 (11)	41 (49)
I understand the importance of regular exercise for my health.	0–2	1.94 (0.24)	0 (0)	78 (94)
I understand the significance of a healthy diet to my health.	0–2	1.88 (0.33)	0 (0)	73 (88)
I know how to search for information about my disease.	0–2	1.76 (0.43)	0 (0)	63 (76)
I know how to get help to reduce my symptoms.	0–2	1.43 (0.59)	4 (5)	40 (48)
I manage my schoolwork (including homework and getting to school).	0–2	1.89 (0.31)	0 (0)	74 (89)
I understand the risks involved in the use of tobacco, alcohol, and drugs.	0–2	1.96 (0.19)	0 (0)	80 (96)
I know where to find information about sexual health.	0–2	1.72 (0.55)	4 (5)	64 (77)
I have support available to help me if I am depressed, tired, or feeling fed up with my disease.	0–2	1.96 (0.19)	0 (0)	80 (96)
Total score	0–26	22.5 (2.5)	0 (0)	8 (10)

**Fig. 2:**
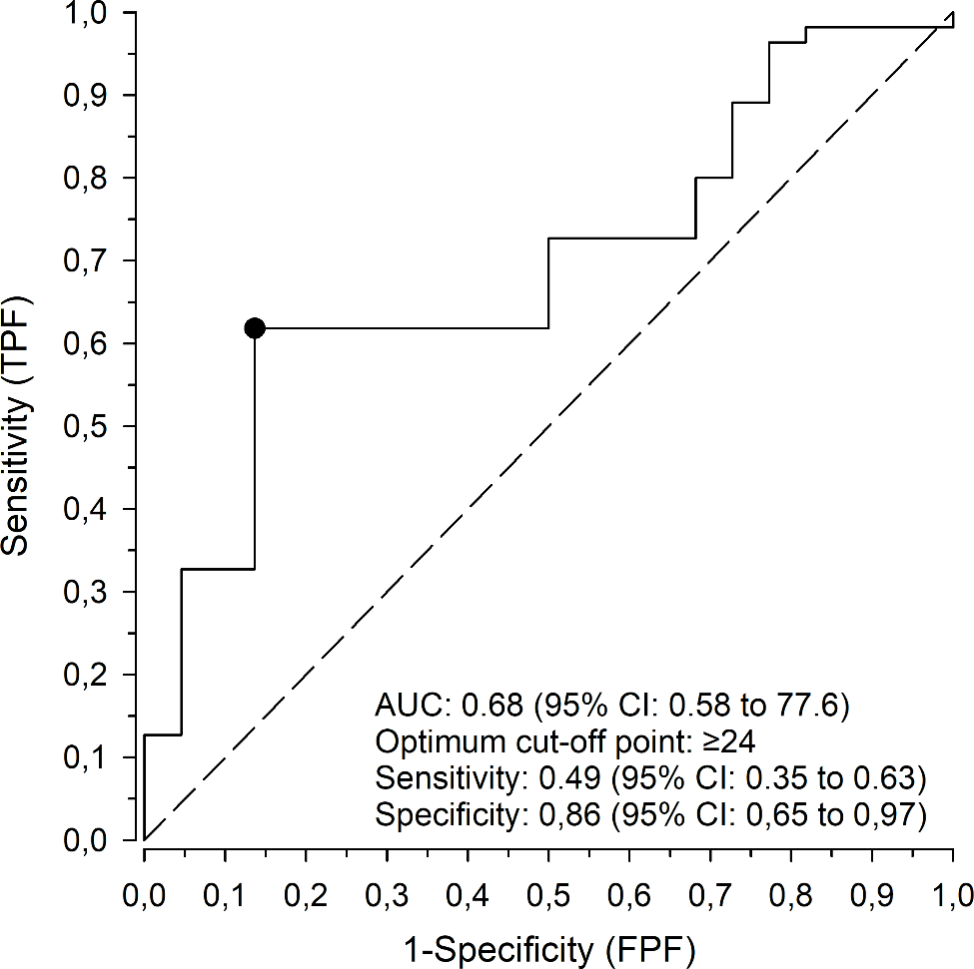
ROC curve for the accuracy of the PETRA-questionnaire in measuring the success of the transition. TPF = the true positive fraction; FNF = the false negative fraction. The 45° diagonal line serves as the reference line since it is the ROC curve of random classification

The cut-off score for a successful transition by ROC-analysis was 24 (OR 6.11 (95% CI: 1.71 to 1.43)) (Fig. 2).

We were able to obtain all the information needed to define the success of the transition for 77 patients. In 55 (71%) patients, the transition was estimated to have been successful.

At the first adult visit, DAS28 was assessed in 58 patients. If the transition was defined as unsuccessful (score < 24), the DAS28 was higher, with a mean of 2.21 (SD = 1.14), and if the transition was defined as successful (score ≥ 24) the DAS28 was lower, with a mean of 1.35 (SD = 0.48), p < 0.001.

## Discussion

In our study, the transition was classified as successful in 71% of the JIA. The main issues behind unsatisfactory results for the transition were poor adherence to medication, inability to comply with scheduled appointments, and the adolescent’s lack of independence at the visits. These are all essential elements of the self-management skills needed for a positive transition process [13]. Other self-management skills include, for example, practical abilities to manage symptoms and administer medications, as well as the skills needed to handle the stress resulting from a chronic condition [13]. During the adolescent years, complex neurodevelopmental processes occur in the brain, and the demands of managing a chronic disease can be overwhelming [22] and present specific challenges during the transition period.

In our study, unsuccessful transitions had an impact on the disease outcome. Although all the transitioned patients had low disease activity as reflected by their DAS28 value, there was, nonetheless, a significant trend showing a relationship between disease activity and success in the transition. To the best of our knowledge, this has not been studied previously in the patients with JIA.

All the patients in our study attended their first adult rheumatological appointment, although, for a few, this was later than that originally scheduled, and they needed extra communication from the adult clinic to ensure their attendance. Drop-out rate from follow-up and care is often used as an estimate for evaluating transition, and low disease activity turned out to be predictive for drop-out [23]. Past studies have presented a discouraging picture of the transition in JIA showing up to half of the transitions being classified as failures [24, 25]. The establishment of our special rheumatology transition clinic at the adult site in 2011, and its protocols, were aimed at preventing loss from follow-up [20]. In our clinic, we transfer patients into the adult clinic at around the age of 16 years, but only patients with active disease and ongoing medication are transferred [19]. Based on the results of our previous study, we transfer around 40% of our adolescent patients [19]. Consequently, in the present study, the clinician’s decision to give the transition questionnaire only to patients with active disease and ongoing medication is in accordance with our transfer practices.

Due to changes in our hospital electronic patient record system, the use of this paper version of the questionnaire remained temporary. However, since doing this study, we have digitalized the questionnaire and separated the different age groups, that is, ages 12–13, 14–15 and over 16 years. The questionnaire can be found in Finnish on an open website funded by university hospitals in Finland (https://www.terveyskyla.fi). Since this digitalization of the questionnaire is relatively recent, we need further research to validate the questionnaire and expand its use to other clinics and to other chronic illnesses as well.

There are several ways to carry out the transition, and worldwide, various transition practices are used [26]. EULAR/PReS has defined standards for transitional care and it provides detailed recommendations about transitioning in JIA [27]. The transition process should start as early as possible, yet be respectful of individual developmental variations [27]. However, even when considering healthcare systems in similar societies, such as the Nordic countries, transition practices vary, as was shown in our previous study [28]. For example, in Finland, the common practice is to transfer adolescents with JIA into an adult clinic at the age of 16, whereas, in the other Nordic countries, the transition age is 18 years [28]. A German observational study regarding transition after kidney transplantation discloses that instead of focusing on the patient’s age during the transition, the focus should be on evaluating their readiness, and the transition should be implemented more flexibly [29]. An ongoing prospective cohort study is exploring transition processes in Finland and Australia, thus introducing potentially interesting cultural differences that may influence transition outcomes [30].

This is a unique study about transition readiness, which evaluates the usefulness of the questionnaire and combines data from both paediatric and adult visits. We have developed a useful and practical tool, the PETRA questionnaire, to evaluate transition readiness among JIA patients. Since our study involves a single paediatric rheumatology centre, there might be challenges in the generalisation and wider use of the questionnaire. More studies and validation are needed to explore the usefulness of the questionnaire, to expand its use more widely and to incorporate other chronic illnesses. The lack of specific data concerning uveitis and the effects of possible mental issues on the transition process can be considered limitations. Further studies that include these elements are essential.

## Conclusion

In this study, we developed a usable instrument for evaluating transition readiness in JIA. Based on our findings, the timing of the transition from paediatric care to the adult site should be flexible, allowing the young person to achieve better readiness, capability, and independence in the care of their chronic disease. The determination of what constitutes a successful transition can help to identify those adolescents who need more profound support and education in improving their self-management skills and thus, enhancing their transition process.

## Data Availability

Data is available upon reasonable request from the study group.

## List of abbreviations

JIA: juvenile idiopathic arthritis
DAS28: Disease Activity Score
sDMARD: synthetic disease-modifying antirheumatic drug
bDMARD: biologic disease-modifying antirheumatic drug
PETRA: pediatric transition readiness to adult care
HUS: hospital district of Helsinki and Uusimaa
HAQ: the Health Assessment Questionnaire
VAS: visual analogy scale
SD: standard deviation
ROC: receiver operating characteristic
AUC: area under curve
OR: odd ratio
IQR: interquartile range
EULAR: European League Against Rheumatism
PReS: Paediatric Rheumatology European Society
ILAR: the International League of Associations for Rheumatology
