# Correction: Transition readiness among finnish adolescents with juvenile idiopathic arthritis

**DOI:** 10.1186/s12969-024-00956-6

**Published:** 2024-01-15

**Authors:** Katriina Mikola, Katariina Rebane, Hannu Kautiainen, Kristiina Aalto

**Affiliations:** 1 New Children’s Hospital, Pediatric Research Center, University of Helsinki and Helsinki University Hospital, Stenbackinkatu 9, 00290 Helsinki, Finland; 2 Primary Health Care Unit Kuopio, Kuopio University Hospital, PohjoisSavo, Finland; 3 Folkhälsan Research Center, Helsinki, Finland

Following publication of the original article [1], we have been notified that the first and last author names have been swapped.

Originally published names: Mikola Katriina, Rebane Katariina, Kautiainen Hannu and Aalto. Kristiina.

Correct name order: Katriina Mikola, Katariina Rebane, Hannu Kautiainen and Kristiina Aalto.

The original article has been corrected.
